# Supplementary material for: Optimization of Chitosan Glutaraldehyde-Crosslinked Beads for Reactive Blue 4 Anionic Dye Removal Using a Surface Response Methodology
Source: Life (Basel). 2021 Jan 25;11(2):85. doi: 10.3390/life11020085 (PMC7912159; doi:10.3390/life11020085)
Supplement: Supplementary file 1 [file life-11-00085-s001.pdf]

life

Supporting information on

Optimization of Chitosan Glutaraldehyde-Crosslinked Beads for Reactive Blue 4 Anionic Dye removal using a Surface Response Methodology

Johanna Galan [1], Jorge Trilleras [2], Paula A. Zapata [3], Victoria A. Arana [1,*] and Carlos David Grande-Tovar [4,*]

[1] Grupo de Investigación Ciencias, Educación y Tecnología - CETIC, Programa de Química, Universidad del Atlántico, *Carrera 30 No 8-49, Puerto Colombia 081008, Colombia*

[2] *Grupo de Compuestos Heterociclicos, Programa de Química, Universidad del Atlántico, Carrera 30 No 8-49, Puerto Colombia 081008, Colombia*

[3] *Grupo de Polímeros, Facultad de Química y Biología, Universidad de Santiago de Chile, USACH, Casilla 40, Correo 33, Santiago 9170020, Chile*

[4] *Grupo de Investigación de Fotoquímica y Fotobiología, Programa de Química, Universidad del Atlántico, Carrera 30 No 8-49, Puerto Colombia 081008, Colombia*

* *Correspondence: victoriaarana@mail.uniatlantico.edu.co (V.A.A.); carlosgrande@mail.uniatlantico.edu.co (C.D.G.T);*

Table S1. *Models of adsorption kinetics.*

Kinetic Model	Model assumptions	Equation	Parameters
**Pseudo- first order**	Adsorption only occurs at fixed sites and does not involve interaction between adsorbed ions.	$q_t = q_e[1 - e^{-k_1 t}]$	q_t: Amount of adsorbate adsorbed over time<br>q_e: Amount of equilibrium adsorption<br>k_1: Pseudo-first order constant<br>t: Equilibrium time
**Pseudo-second order**	Adsorption only occurs at specific sites, and there is an interaction between adsorbed ions.	$q_t = \frac{K_2 q_e^2 t}{1 + K_2 q_e t}$	K_2: Pseudo-second order constant
**Elovich equation**	Active adsorption sites are heterogeneous and therefore	$q_t = \left(\frac{1}{\beta}\right) \ln(\alpha.\beta) + \left(\frac{1}{\beta}\right) \ln{(t)}$	α: Elovich equation constant

	exhibit different activation energies.		β: exponent in Elovich's equation
**Intraparticular difusión**	The adsorption rate could be limited by the size of the adsorbate molecule, its concentration, and the affinity it exhibits for the adsorbent.	$q_t = k_{id}\sqrt{t} + C$	k_{id}: Constante de difusión<br>C: Intercept

Source: adapted from [1]

Table S2. *Adsorption isotherm models.*

Isotherm	Model assumptions	Nonlinear equation	Parameters
**Langmuir**	It assumes monolayer adsorption (the adsorbed layer is one molecule thick), and in turn, adsorption can only occur at a finite (fixed) number of defined localized sites, which are identical and equivalent.	$q_e = \frac{K_L q_m C_e}{1 + K_L C_e}$	q_e: Adsorption capacity at equilibrium.<br>q_m: Maximum adsorption capacity.<br>C_e: Adsorbate concentration at equilibrium.<br>K_L: Adsorption constant at equilibrium
**Freundlich**	It assumes multilayer adsorption, with non-uniform adsorption heat distribution on the adsorbent surface.	$q_e = K_F (C_e)^{\frac{1}{n}}$	K_F: an indicator of adsorption capacity.<br>$\frac{1}{n}$: Intensity of adsorption on the heterogeneous surface.

**Temkin**	Proposes into account the effects of the interaction of the adsorbate and the adsorbing species. By ignoring the shallow and large concentration values, the model assumes that the heat of adsorption (a function of temperature) of all the molecules in the layer would decrease linearly rather than logarithmically with coverage due to adsorbate–adsorbent interactions.	$q_e = \frac{RT}{b_T} ln(K_T C_e)$	K_T: Temkin's constant considers adsorbent-adsorbate interactions and corresponds to the maximum binding energy.<br>b_T: constant related to the heat of adsorption.<br>R: universal gas constant.<br>T: absolute temperature.

Source: adapted from [2]

References

1. Largitte, L.; Pasquier, R. A review of the kinetics adsorption models and their application to the adsorption of lead by an activated carbon. *Chem. Eng. Res. Des.* **2016**, *109*, 495–504, doi:10.1016/j.cherd.2016.02.006.

2. Aljeboree, A.M.; Alshirifi, A.N.; Alkaim, A.F. Kinetics and equilibrium study for the adsorption of textile dyes on coconut shell activated carbon. *Arab. J. Chem.* **2014**, *10*, S3381–S3393.
